# Generative artificial intelligence in forensic medicine: a pilot study on AI-simulated medico-legal reports in healthcare liability cases

**DOI:** 10.1007/s00414-026-03820-2

**Published:** 2026-05-02

**Authors:** Federica Ministeri, Massimiliano Esposito, Martina Francaviglia, Lucio Di Mauro, Grazia Giulia Pantè, Monica Salerno, Cristoforo Pomara, Francesco Sessa

**Affiliations:** 1 Department of Medical, Surgical and Advanced Technologies “G.F. Ingrassia”, University of Catania, Catania, 95121 Italy; 2 Faculty of Medicine and Surgery, “Kore” University of Enna, Enna, 94100 Italy; 3 Department of Psychology and Health Sciences, Faculty of Human Sciences, Education, and Sports, Pegaso Telematic University, Naples, 80143 Italy

**Keywords:** Artificial intelligence (AI), Large language models, Forensic medicine, Medico-legal evaluation, Healthcare liability, Generative AI

## Abstract

**Background:**

Healthcare liability litigation has increased substantially, highlighting the need for structured and evidence-based medico-legal evaluations. While generative artificial intelligence (AI) has been extensively studied in clinical medicine, its application in medico-legal consultancy remains limited.

**Objective:**

To assess whether two customized GPT-based models, simulating opposing medico-legal perspectives (“patient-oriented” and “hospital-oriented”), can analyze real clinical documentation and generate reports comparable to those produced by human experts.

**Methods:**

Ten real healthcare liability cases were retrospectively analyzed. For each case, complete anonymized clinical records and human-authored medico-legal reports were available. Two GPT-based models were configured using domain-specific instructions and authoritative medico-legal references, without retraining. Both models generated simulated reports from identical documentation. Outputs were evaluated using structured qualitative scores assessing argumentative coherence, clinical synthesis, bibliographic accuracy and relevance, and concordance of permanent impairment estimates.

**Results:**

No significant difference in overall argumentative coherence was observed between models (Wilcoxon test, *p* = 0.844). Orientation-consistent trends emerged after stratification by liability status. Agreement was substantial for clinical synthesis (Cohen’s κ = 0.804) but slight for permanent impairment estimates (κ = 0.167). The patient-oriented model produced significantly more fabricated or non-verifiable references (OR = 3.74; *p* = 0.0065), while citation relevance did not differ significantly between models (*p* = 0.195).

**Conclusions:**

Generative AI can simulate divergent medico-legal reasoning and rapidly produce structured reports. However, expert human oversight remains essential, particularly for source verification and medico-legal interpretation.

**Supplementary Information:**

The online version contains supplementary material available at 10.1007/s00414-026-03820-2.

## Introduction

Healthcare liability litigation has progressively increased over recent decades, reflecting growing patient expectations, rising clinical complexity, and enhanced judicial scrutiny of adverse healthcare events [1–4]. Within this context, medico-legal experts play a pivotal role at the intersection between medicine and law, being required to assess the appropriateness of healthcare conduct, determine causal relationships between alleged errors and harm, and quantify biological damage with scientific rigor and impartiality [5]. Despite the centrality of this function, medico-legal evaluations inherently involve interpretative judgment, often resulting in substantial variability between opposing expert opinions [6–8].

In response to this complexity, modern medico-legal practice has increasingly emphasized evidence-based reasoning, methodological transparency, and explicit reference to clinical guidelines and scientific literature valid at the time of the alleged event [9]. In Italy, this approach has been formally reinforced by Law no. 24/2017 (Gelli–Bianco Law), which strengthened the role of clinical guidelines and good practices in healthcare liability assessments and promoted structured risk management strategies [10]. Similar trends are observed across other legal systems, where courts and healthcare institutions increasingly expect medico-legal opinions to be grounded in verifiable scientific evidence rather than subjective expert interpretation alone [11–13].

Against this backdrop, variability in medico-legal reasoning remains a critical issue, particularly in adversarial contexts such as healthcare litigation. Differences in interpretative frameworks, selective use of literature, and divergent weighting of clinical facts can lead to markedly different conclusions even when experts analyze the same documentation. This variability represents not only a methodological challenge but also a potential source of uncertainty for judicial decision-making and institutional risk management [6, 14, 15].

In parallel, artificial intelligence (AI), and specifically large language models (LLMs) based on generative architectures, has rapidly expanded its presence in healthcare, demonstrating the ability to analyze complex textual data, synthesize information, and generate structured narratives [16–18]. While these systems have been investigated in several clinical domains, including documentation support, diagnostic assistance, and risk prediction, their application within forensic medicine and medico-legal consultancy remains largely unexplored. This gap is particularly relevant given that medico-legal practice is primarily based on the analysis of clinical documentation and on the evaluation of relevant scientific literature and clinical guidelines [19].

To date, no experimental studies have evaluated whether generative AI systems can simulate medico-legal reasoning by analyzing real clinical documentation and producing structured medico-legal reports comparable to those authored by human experts. Moreover, the potential of AI to reproduce divergent argumentative perspectives, such as those typically adopted by experts acting on behalf of patients or healthcare institutions, has not been systematically investigated.

The present study addresses this gap by exploring the use of two customized GPT-based models configured to simulate opposing medico-legal perspectives in real cases of alleged healthcare liability. By comparing AI-generated reports with human-authored medico-legal evaluations, this study aims to assess the coherence, reliability, and limitations of AI-simulated medico-legal reasoning, and to explore its potential role as a preliminary support tool in contemporary forensic practice, rather than as a substitute for human expertise.

## Materials and methods

### Study design, setting, and case selection

This pilot study was conducted at the Institute of Legal Medicine of the University of Catania and was designed as a retrospective, qualitative, comparative analysis. A retrospective analysis of medico-legal litigation cases examined at the Institute between 2020 and 2025 was performed. The initial dataset consisted of 350 cases, which were subsequently assessed for eligibility according to the predefined selection criteria.

Cases were included if all of the following criteria were met:

1. Availability of a medico-legal report issued by the Institute of Legal Medicine on behalf of the involved healthcare institution;
2. Availability of a medico-legal report prepared on behalf of the patient or a detailed compensation claim supported by medico-legal arguments;

Cases were excluded if:

1. The patient died during the healthcare episode (claims involving death, for which no estimation of permanent biological damage was applicable);
2. Incomplete or unavailable clinical documentation related to the healthcare episode under dispute.

After application of these criteria, 10 cases were included in the final analysis. Five cases had been previously classified as non-liability cases by the Institute of Legal Medicine, resulting in dismissal of compensation claims, while the remaining five cases involved confirmed healthcare liability with an associated estimate of permanent biological impairment.

All documentation was fully anonymized prior to analysis.

### Configuration of customized GPT models

Two customized GPT-based models were developed using the ChatGPT platform (OpenAI) [20] to simulate opposing medico-legal perspectives typically encountered in healthcare litigation:

1. A patient-oriented model (“AI in Defense of the Patient”);
2. A hospital-oriented model (“AI in Defense of the Hospital”).

In this study, the term GPT refers to a generative large language model capable of processing and generating natural language. The models were configured through prompt-engineering and document injection, by uploading curated medico-legal reference materials and model-specific instructional files.

Both models were provided with the same core set of authoritative medico-legal sources addressing healthcare liability, causal assessment, and evaluation of biological damage (*Supplementary Material **1**)*. In addition, each model received a dedicated set of written instructions, designed to define its evaluative stance and guide the structure and content of the generated medico-legal reports (*Supplementary Material **2**-**3**-**4**)*.

The models were used in their default configuration without parameter tuning (e.g., temperature or top-p), and no fine-tuning or retraining procedures were applied. The outputs were entirely determined by the provided documentation and predefined instructional prompts.

The patient-oriented model was supplied with instructions explicitly directing it to critically analyze clinical documentation, identify potential deviations from good clinical practice, and support hypotheses of healthcare liability when scientifically plausible.

Conversely, the hospital-oriented model received instructions aimed at emphasizing adherence to clinical guidelines and standards of care, contextualizing adverse events, and excluding or mitigating liability when justified by the available evidence.

Both models were instructed to produce formally structured medico-legal reports, including sections on clinical documentation, clinical course, medico-legal evaluation, conclusions, and bibliography. When generating bibliographic references, models were required to rely exclusively on clinical guidelines and scientific literature indexed in PubMed or Scopus and valid at the time of the healthcare event.

### 2.3 Generation of AI-simulated medico-legal reports

For each of the selected cases, identical anonymized clinical documentation (medical records, emergency department reports, discharge summaries, diagnostic findings, and follow-up data) was provided to both GPT models. No interactive prompts, clarifications, or corrective inputs were introduced during report generation.

Each model independently generated a simulated medico-legal report consistent with its predefined evaluative orientation. This approach was adopted to avoid operator-induced bias and to assess the autonomous performance of the configured models.

### Evaluation framework

AI-generated reports were evaluated by a single medico-legal expert, using a structured, multi-level qualitative framework.

1. Comparison with human-authored medico-legal reports:

For each case, the medico-legal reports generated by the artificial intelligence models were directly compared with the corresponding reports authored by human medico-legal experts. Two qualitative scores were assigned:

- *Argumentative coherence and concordance* (1–10), assessing alignment with the conclusions and reasoning of human experts;
- *Quality of clinical synthesis* (1–3), evaluating clarity, completeness, and accuracy in reconstructing the clinical course.

2. Evaluation of bibliographic references:

Each scientific reference generated by the models was assessed according to a predefined grid evaluating:

- Existence of the cited article;
- Correctness of authorship, journal, and publication year;
- Validity of the DOI or bibliographic link;
- Relevance to the specific clinical case (scored on a 0–3 scale).

Fabricated or non-existent references were assigned a score of zero across all categories.

3. Evaluation of permanent biological impairment estimates:

In cases with confirmed healthcare liability, AI-generated estimates of permanent biological impairment were compared with those reported in human medico-legal evaluations. Concordance was scored on a 0–3 scale (non-concordant to highly concordant).

This evaluation framework enabled a comprehensive assessment of the coherence, reliability, and medico-legal consistency of AI-generated outputs.

The evaluation framework was specifically designed for this exploratory pilot study and was not previously validated. Therefore, the scoring system should be interpreted as an exploratory qualitative tool rather than a standardized measurement instrument.

### Statistical analysis

Given the small sample size and the ordinal nature of the scoring scales, we performed an exploratory non-parametric analysis. Paired comparisons of argumentative coherence scores between the hospital-oriented and patient-oriented GPT models were assessed using the Wilcoxon signed-rank test overall and stratified by liability status (cases without confirmed malpractice vs. cases with confirmed malpractice). Effect sizes for paired comparisons were reported as rank-biserial correlation (r_rb), computed from the signed-rank decomposition.

Inter-model agreement on clinical synthesis scores (1–3) and on impairment estimates (0–3, liability cases only) was quantified using Cohen’s κ with unweighted categories. Fabricated or non-verifiable references were operationally defined as citation entries scoring 0 simultaneously on all four formal dimensions (authorship, journal, year, DOI/link). The proportion of fabricated references was compared between models using Fisher’s exact test on 2 × 2 contingency tables. To compare the ordinal distributions of citation relevance (0–3) between models, we applied a Mann–Whitney U test (noting that, for two groups, it is equivalent to a Jonckheere–Terpstra trend test). A two-sided α = 0.05 was adopted, and all analyses were interpreted as exploratory.

## Results

The evaluation of AI-generated medico-legal reports was conducted according to the predefined multi-level qualitative framework described in the Methods section. Results are presented with reference to three main domains: (1) coherence and quality of medico-legal reasoning, (2) quality of clinical synthesis, (3) characteristics and quality of bibliographic references, and (4) concordance of permanent biological impairment estimates in liability-confirmed cases.

Descriptive statistics were generated and stratified by case category (with vs. without confirmed healthcare liability), as well as aggregated across the entire dataset. This framework enabled an assessment of the overall reliability of AI-generated reports, the functional consistency of the two models, and the scientific appropriateness of the citations automatically inserted by the AI.

### Comparative analysis of medico-legal reasoning

Across the 10 selected cases, AI-generated medico-legal reports were compared with corresponding human-authored evaluations. Argumentative coherence and concordance were assessed using a 1–10 qualitative score. Detailed case-by-case scoring is provided in *Supplementary Material **5*.

In the five cases classified as non-liability, the hospital-oriented GPT model achieved higher coherence scores, with a mean value of 8.0, compared with a mean score of 5.4 for the patient-oriented model.

Conversely, in the five cases involving confirmed healthcare liability, the patient-oriented GPT demonstrated higher coherence, with a mean score of 5.2, whereas the hospital-oriented model showed a lower mean score of 3.6.

**Fig. 1:**
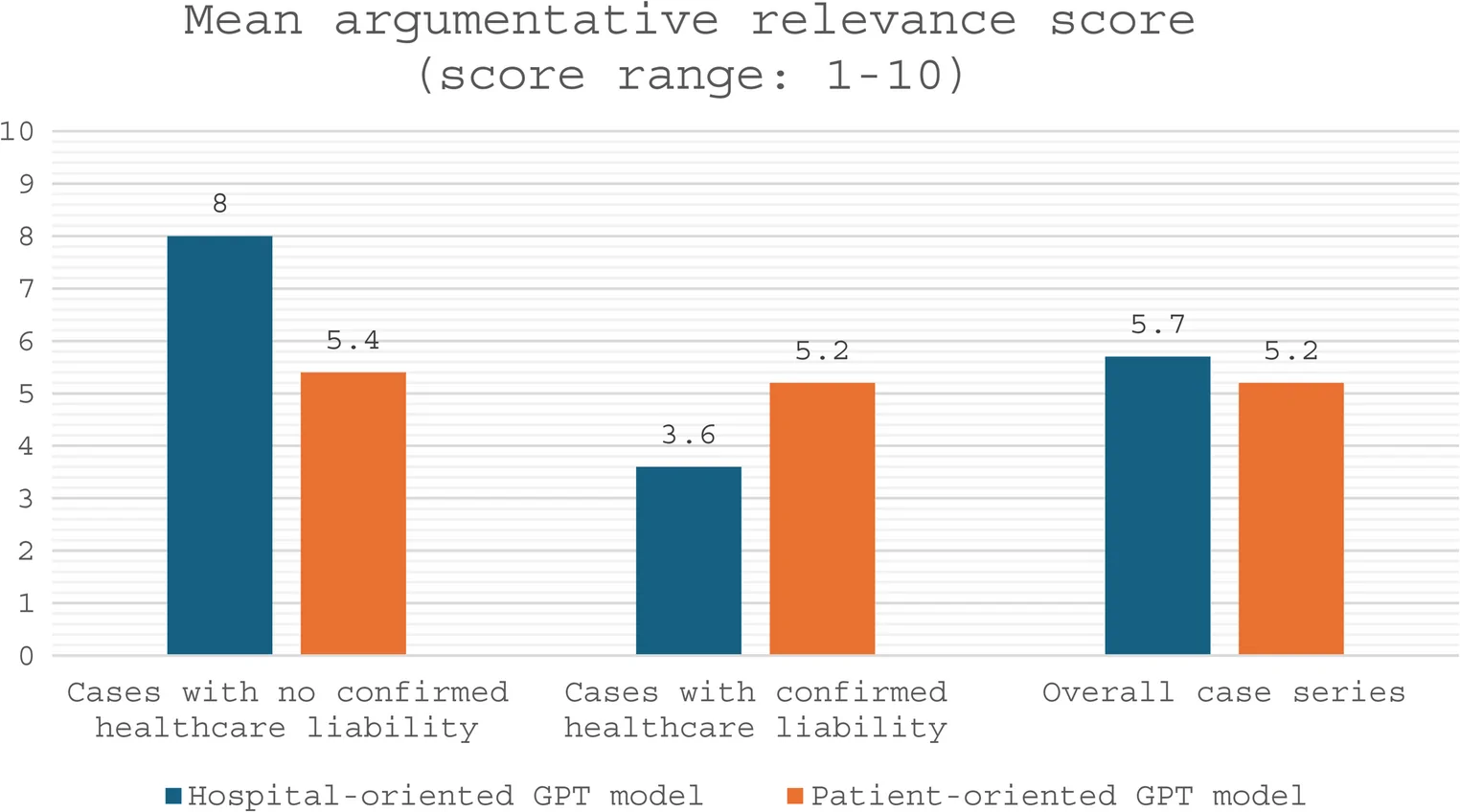
Qualitative statistical analysis of the argumentative coherence of AI-generated medico-legal reports (score range: 1–10)

Considering the entire case series, the overall mean coherence score was 5.7 for the hospital-oriented model and 5.2 for the patient-oriented model. These results indicate a consistent alignment between each model’s predefined evaluative orientation and the medico-legal conclusions reached in human-authored reports (Fig. 1).

### Quality of clinical synthesis

The quality of clinical synthesis generated by both GPT models was evaluated using a 1–3 scale, focusing on clarity, completeness, and accuracy in reconstructing the clinical course. Detailed case-by-case scoring is provided in *Supplementary Material **6*.

**Fig. 2:**
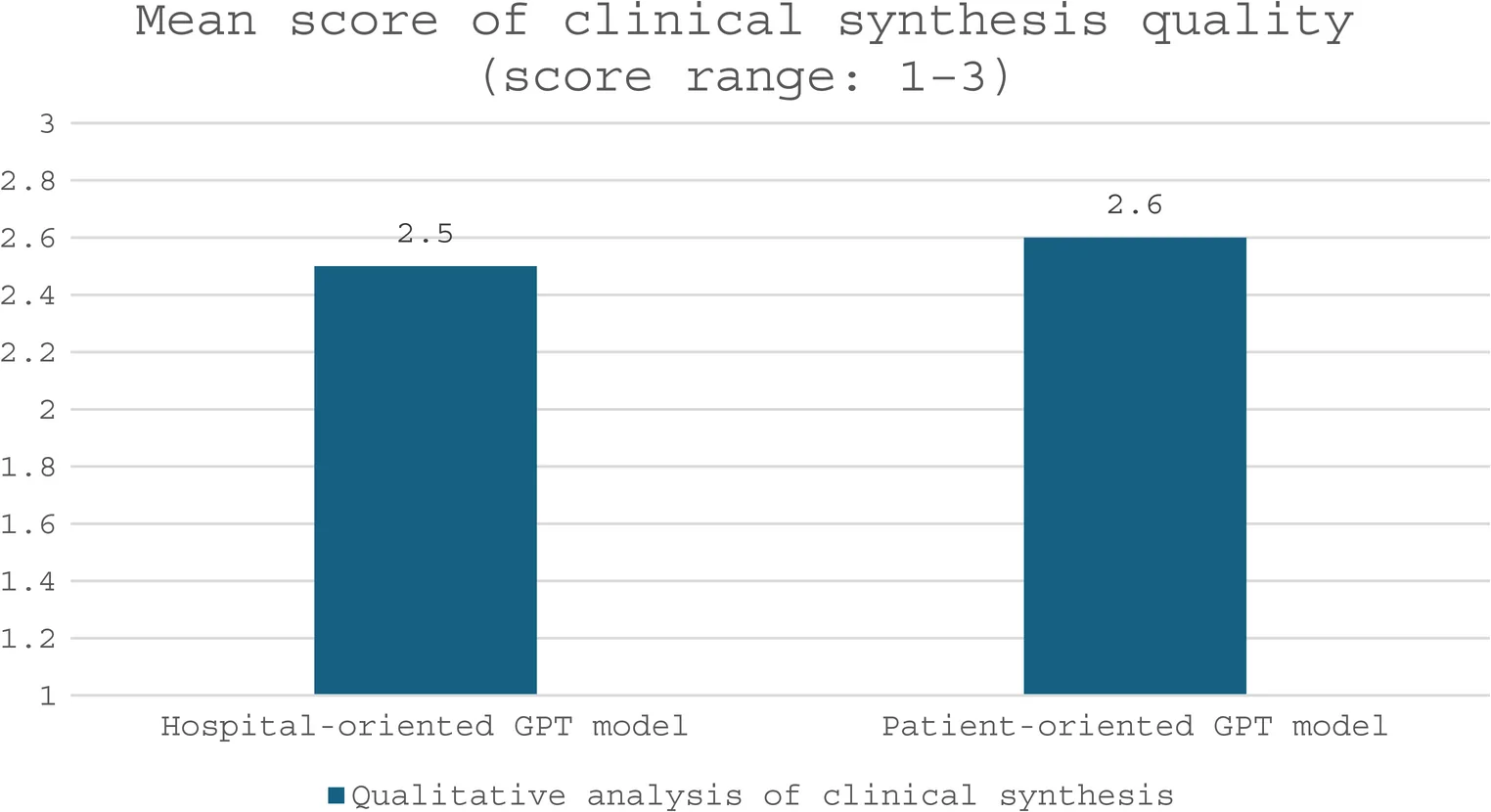
Qualitative statistical analysis of AI-generated clinical synthesis (score range: 1–3)

The patient-oriented model achieved a mean synthesis score of 2.6, while the hospital-oriented model achieved a mean score of 2.5. Scores were generally stable across cases and did not differ substantially between liability and non-liability groups. Overall, both models demonstrated a comparable ability to summarize and organize clinical documentation in a coherent manner (Fig. 2).

### Analysis of bibliographic references

The bibliographic references generated by the two GPT models were analyzed both quantitatively and qualitatively and compared with those reported in human-authored medico-legal reports. Detailed case-by-case scoring is provided in *Supplementary Material **7* and *8*.

**Fig. 3:**
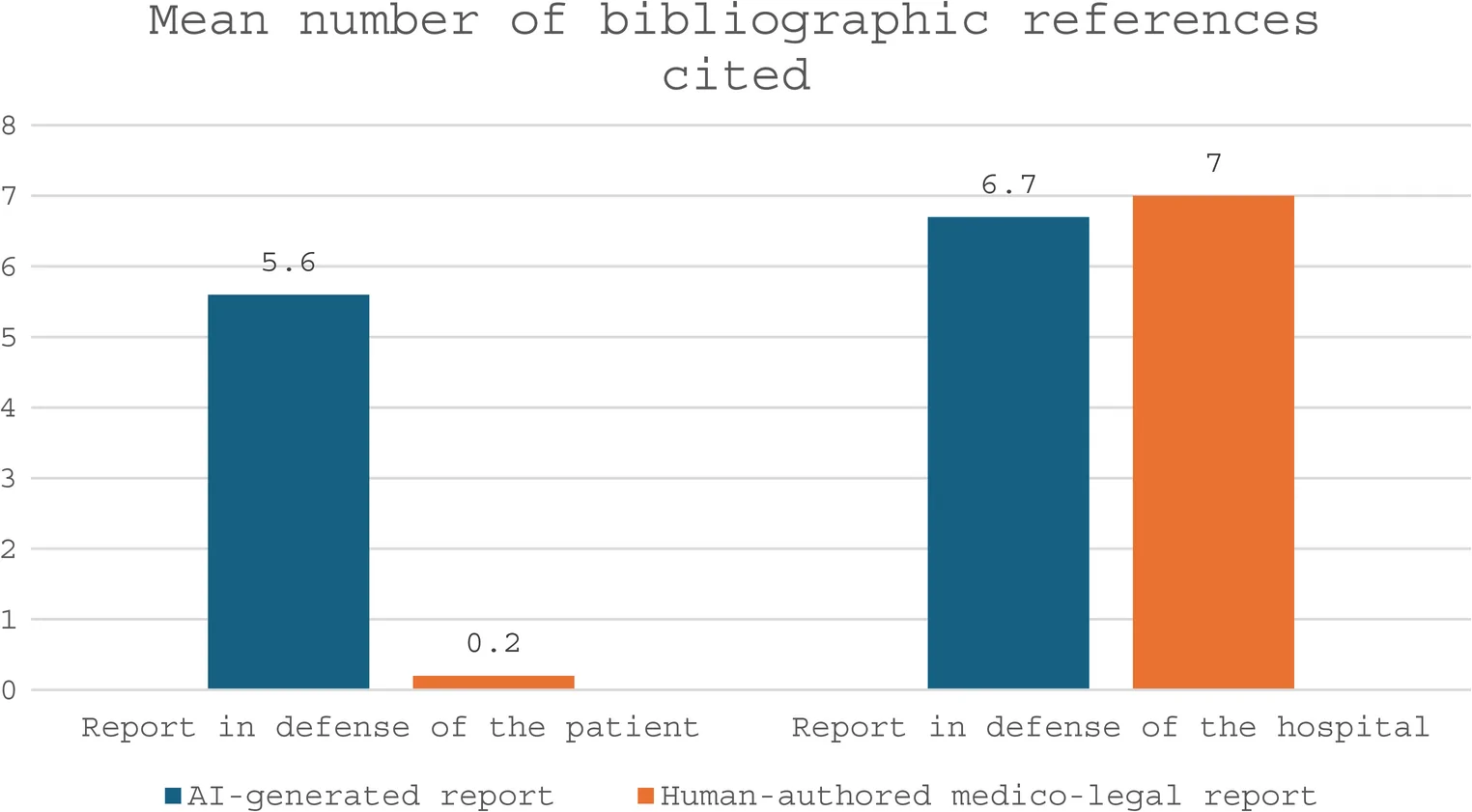
Quantitative statistical analysis of bibliographic sources

The mean number of references generated per case was 5.6 for the patient-oriented model and 6.7 for the hospital-oriented model. In comparison, human-authored reports included a mean of 0.2 references per case in patient-side reports and 7 references per case in hospital-side reports (Fig. 3).

**Fig. 4:**
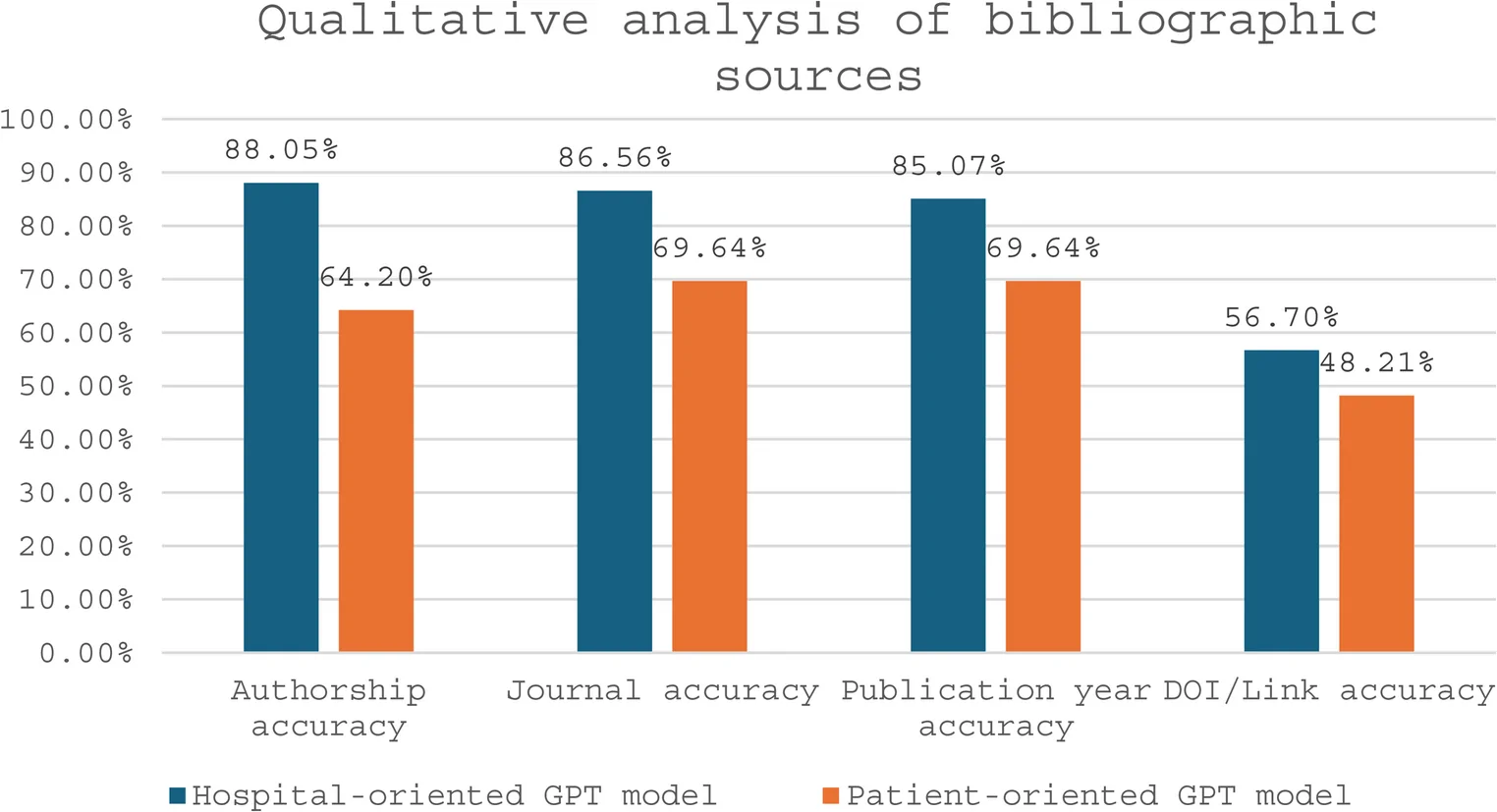
Qualitative statistical analysis of the formal accuracy of authorship, journal, publication year, and DOI/link of AI-generated references

Qualitative analysis of AI-generated references revealed differences in formal accuracy between the two models. The hospital-oriented GPT produced references with correct authorship in 88.05% of cases, correct journal attribution in 86.56%, and correct publication year in 85.07%. The corresponding values for the patient-oriented model were lower, with authorship accuracy of 64.2%, journal correctness of 69.64%, and publication year accuracy of 69.64%. Valid DOI or bibliographic link availability was observed in 56.7% of references generated by the hospital-oriented model and in 48.21% of those generated by the patient-oriented model (Fig. 4).

Regarding relevance to the specific clinical cases, references generated by the patient-oriented model were classified as highly relevant (score 3) in 48.2% of cases, relevant (score 2) in 7.14%, partially relevant (score 1) in 5.35%, and non-relevant (score 0) in 39.2%. In contrast, the hospital-oriented model produced a higher proportion of relevant references, with 49.25% classified as highly relevant, 20.89% as relevant, 13.4% as partially relevant, and 16.41% as non-relevant. The mean relevance score (maximum 3) was 1.64 for the patient-oriented model and 2.02 for the hospital-oriented model.

**Fig. 5:**
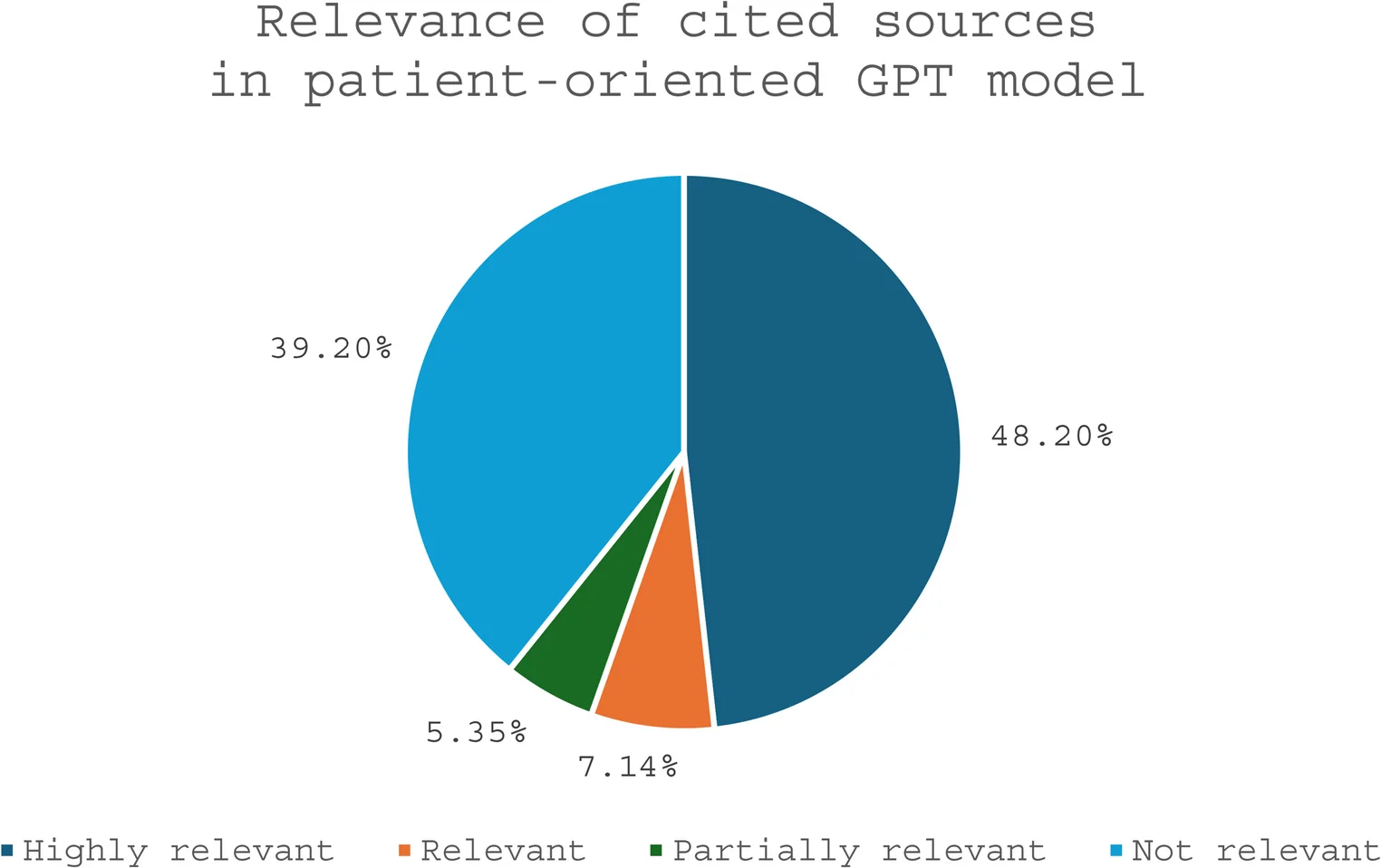
Qualitative statistical analysis of the relevance of bibliographic sources cited by the patient-oriented GPT model

**Fig. 6:**
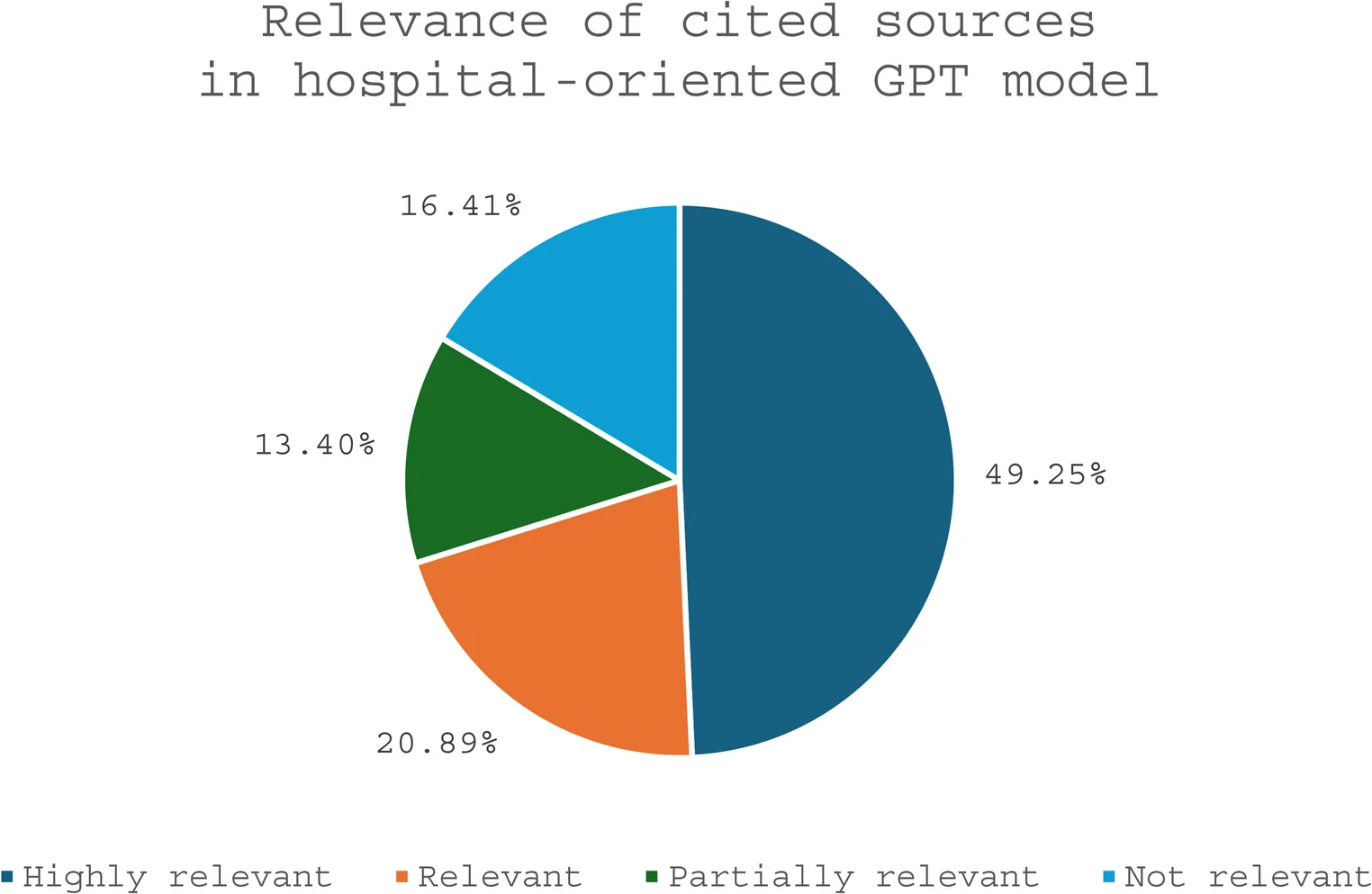
Qualitative statistical analysis of the relevance of bibliographic sources cited by the hospital-oriented GPT model

Across both models, a subset of generated references was found to be non-existent or unverifiable, resulting in a score of zero across all evaluated parameters (Figs. 5 and 6).

### Evaluation of permanent biological impairment estimates

Assessment of permanent biological impairment estimates was performed exclusively in the five cases with confirmed healthcare liability. In these cases, the original medico-legal reports included impairment percentages provided independently by the Hospital’s consultants and the Patient’s experts. Detailed case-by-case scoring is provided in *Supplementary Material **9*.

The patient-oriented GPT produced impairment estimates rated as highly concordant with human expert evaluations (score 3) in four out of five cases (80%), with one non-concordant estimate.

The hospital-oriented GPT produced one highly concordant estimate (20%), one concordant estimate (20%), and three non-concordant estimates (60%).

**Fig. 7:**
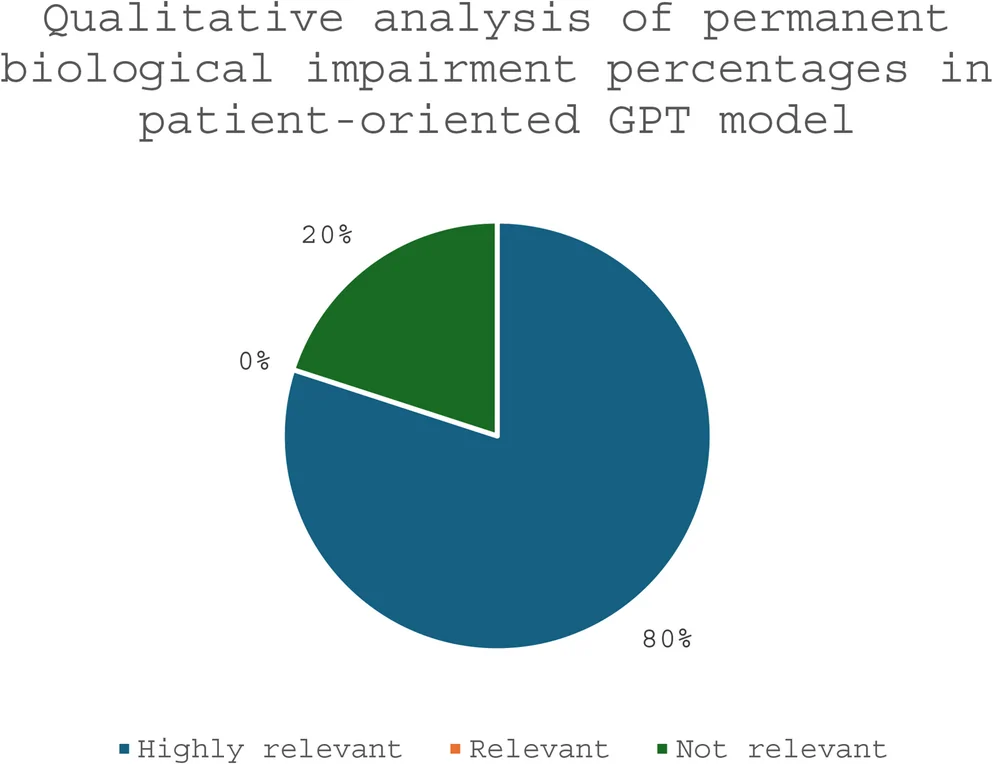
Qualitative statistical analysis of biological impairment percentages generated by the patient-oriented GPT model

**Fig. 8:**
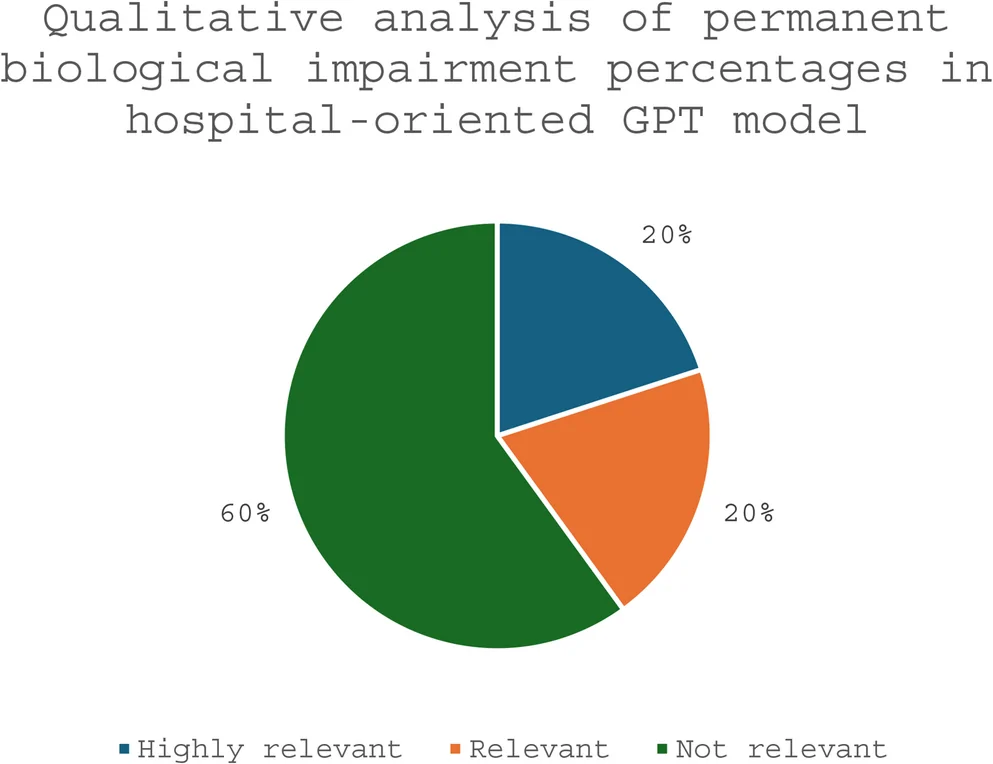
Qualitative statistical analysis of biological impairment percentages generated by the hospital-oriented GPT model

These findings indicate a higher level of agreement between the patient-oriented model and human expert assessments in cases involving established liability (Figs. 7 and 8).

### Statistical findings

Across all ten cases, the paired comparison of argumentative coherence scores showed no overall difference between the patient‑ and hospital‑oriented models (Wilcoxon signed‑rank: *n* = 8 non‑tied pairs, *p* = 0.844, r₍rb₎ = −0.08). Stratification by liability status revealed the expected orientation effect: in non‑liability cases (cases 1–5), the hospital‑oriented model tended to score higher (Wilcoxon: *n* = 5, *p* = 0.438, r₍rb₎ = −0.40), whereas in liability cases (cases 6–10) the patient‑oriented model tended to score higher (Wilcoxon: *n* = 3, *p* = 0.500, r₍rb₎ = +0.67).

Inter‑model agreement on clinical synthesis (1–3) was substantial‑to‑almost perfect (Cohen’s κ = 0.804), confirming consistent reconstruction of the clinical course by both models. Conversely, agreement on impairment estimates in liability cases (five cases) was slight (Cohen’s κ = 0.167), reflecting the known sensitivity of quantitative impairment appraisal to the evaluative stance.

At the citation level (123 AI‑generated references parsed), the patient‑oriented model produced 17/56 fabricated or non‑verifiable citations versus 7/67 for the hospital‑oriented model; the difference was statistically significant (Fisher’s exact test: odds ratio = 3.74, *p* = 0.0065). In contrast, the ordinal relevance of citations (0–3) did not differ significantly between models (Mann–Whitney U = 1639, *p* = 0.195, *r* = 0.109; small effect), suggesting that while formal verifiability is uneven, topical pertinence is broadly comparable.

## Discussion

This pilot study represents an initial empirical assessment of the ability of generative artificial intelligence to simulate medico-legal reasoning in real cases of alleged healthcare liability. The findings show that GPT-based models, when configured with divergent evaluative orientations, are capable of producing structured medico-legal reports from identical clinical documentation, with outputs that vary predictably according to the assigned perspective. The objective was to evaluate whether generative artificial intelligence models, configured to adopt predefined medico-legal perspectives, could analyze real clinical documentation and generate simulated medico-legal reports comparable to those produced by human forensic experts in the context of healthcare liability.

The overall comparison of argumentative coherence revealed no statistically significant difference between the patient-oriented and hospital-oriented models (Wilcoxon signed-rank test, *p* = 0.844). This finding suggests that, when considered globally, both models achieve a comparable level of alignment with human-authored medico-legal reports.

When cases were stratified by liability status, a directionally consistent orientation effect emerged. In non-liability cases, the hospital-oriented model tended to achieve higher coherence scores, whereas in liability-confirmed cases the patient-oriented model tended to perform better. Although these differences did not reach statistical significance (*p* = 0.438 and *p* = 0.500, respectively), effect size estimates indicated a moderate-to-large directional trend, particularly in liability cases (r₍rb₎ = +0.67). This pattern is consistent with the predefined evaluative conditioning of the models and demonstrates that role assignment systematically influences AI-generated medico-legal reasoning, even in the absence of statistically significant differences due to limited sample size [21, 22].

Inter-model agreement analyses further delineate the functional domains in which generative AI performs more reliably. Agreement between models was substantial to almost perfect for clinical synthesis (Cohen’s κ = 0.804), confirming that both systems consistently reconstructed the clinical course from identical documentation. This finding confirms that generative AI is particularly effective in organizing heterogeneous clinical records into coherent narratives, a task that represents a substantial component of routine medico-legal activity.

However, this strength must be interpreted cautiously. In cases characterized by complex clinical trajectories, prolonged timelines, or subtle medico-legal nuances, the models occasionally failed to capture elements that would be considered critical by human experts, such as borderline deviations from best practice, ambiguous temporal relationships, or context-dependent clinical decisions. This limitation reflects the models’ reliance on textual pattern recognition rather than on conceptual or causal understanding, and it reinforces the necessity of expert interpretation when AI-generated summaries are used in medico-legal contexts.

Conversely, agreement on permanent biological impairment estimates was slight (Cohen’s κ = 0.167), highlighting a critical limitation. The estimation of impairment is inherently evaluative and normative, requiring integration of clinical findings, causal reasoning, functional assessment, and medico-legal standards. The low concordance observed confirms that AI-generated quantitative outputs in this domain are highly sensitive to evaluative orientation and cannot be considered reliable without expert oversight.

The statistical analysis of bibliographic outputs revealed one of the most clinically relevant findings of the study. The patient-oriented model generated a significantly higher proportion of fabricated or non-verifiable citations compared with the hospital-oriented model (OR = 3.74; *p* = 0.0065). This statistically significant difference confirms that bibliographic reliability is not uniform across role-conditioned models and may be influenced by the argumentative stance imposed during configuration. In contrast, no significant difference was observed in citation relevance between models (Mann–Whitney U test, *p* = 0.195), indicating that topical pertinence and formal verifiability represent distinct dimensions of bibliographic quality.

From a medico-legal perspective, this distinction is crucial. While AI systems may be capable of identifying thematically appropriate literature, the generation of inaccurate or non-existent references poses a substantial risk in forensic contexts, where evidentiary reliability and traceability are mandatory. In judicial contexts, the validity, traceability, and verifiability of scientific references are essential prerequisites for evidentiary reliability. The generation of non-existent or inaccurate citations by AI systems represents a critical limitation, as it may compromise the credibility of medico-legal reports and potentially affect judicial decision-making. In adversarial legal settings, where expert reports are subject to scrutiny by opposing parties and courts, even a single fabricated reference may undermine the overall reliability of the document. Therefore, strict human validation of all AI-generated bibliographic content is mandatory [14, 15, 23, 24].

Taken together, the results of this study indicate that generative AI may have a role in selected preparatory phases of medico-legal activity, such as the organization of clinical documentation, the drafting of structured summaries, and the simulation of alternative argumentative perspectives. However, the limitations observed, particularly with respect to interpretative depth of clinical data and bibliographic reliability, preclude autonomous use in medico-legal decision-making [22, 25, 26].

The integration of generative AI into forensic practice should be governed by clear frameworks emphasizing transparency, traceability, and mandatory human oversight, ensuring that technological support enhances efficiency without undermining professional responsibility [27, 28].

These findings also raise important ethical and legal considerations, particularly regarding responsibility, accountability, and transparency in AI-assisted medico-legal evaluations. The use of non-transparent generative systems in forensic contexts may challenge the requirement for explainability in legal proceedings, highlighting the need for clear regulatory frameworks.

### Strengths and Limitations

As a pilot investigation, this study has several limitations. The sample size was small, reflecting the exploratory nature of the project, and included only cases with full documentation and clear expert reports. Additionally, the study relied on a single AI platform and a specific set of customized instructions. Different models or training materials could yield divergent outputs. All qualitative evaluations were performed by a single medico-legal expert, and no inter-rater reliability assessment was conducted. This represents a significant methodological limitation, particularly in medico-legal research, where interpretative variability between experts is well documented. Future studies should include multiple independent evaluators and formal agreement analysis to strengthen the robustness of the findings.

Finally, the models were not tested in dynamic or interactive contexts. However, this aspect also represents a methodological strength of the study, as the absence of any real-time interaction or iterative prompting ensured that the AI systems operated autonomously, relying exclusively on the preloaded instructions and source materials. This approach allowed for a more objective assessment of the models’ intrinsic reasoning and generative capabilities, independent of human guidance during the report generation process.

## Conclusions

This pilot study provides the first experimental evidence that generative artificial intelligence, when appropriately configured with domain-specific instructions, can simulate divergent medico-legal reasoning in real cases of alleged healthcare liability. These findings should be interpreted with caution given the exploratory design and limited sample size. The ability of the customized GPT models to generate coherent, structured and perspective-aligned medico-legal reports within seconds underscores the potential value of AI as an assistive tool in the early analytical stages of medico-legal practice.

The differential performance observed between the patient-oriented and hospital-oriented models highlights the extent to which AI-driven outputs are influenced by role conditioning, reinforcing the need for carefully designed governance frameworks and strict oversight by qualified experts. While the models demonstrated strong capabilities in clinical synthesis and produced impairment estimates that were often concordant with human evaluations, the presence of hallucinated or formally inaccurate citations confirms that AI cannot independently ensure evidentiary reliability.

Taken together, these findings suggest that generative AI should be considered a promising adjunct for organizing documentation, exploring alternative argumentative perspectives, and supporting preliminary medico-legal analyses, but not as a substitute for human expertise. The medico-legal domain requires interpretative nuance, contextual understanding, and ethical accountability that remain beyond the reach of current AI systems.

Future work should expand the evidence base by incorporating larger and more diverse case series, integrating jurisdiction-specific medico-legal frameworks, and developing safeguards that mitigate the risks associated with automated citation generation and interpretative errors. As generative AI continues to evolve, interdisciplinary collaboration among forensic physicians, data scientists, and legal scholars will be essential to ensure that technological innovation enhances the rigor, transparency, and fairness of medico-legal assessments.

## Electronic Supplementary Material

Below is the link to the electronic supplementary material.

Supplementary Material 1
