# Supplementary material for: Generative artificial intelligence in forensic medicine: a pilot study on AI-simulated medico-legal reports in healthcare liability cases
Source: Int J Legal Med. 2026 May 2;140(5):3393–403. doi: 10.1007/s00414-026-03820-2 (PMC13499736; doi:10.1007/s00414-026-03820-2)
Supplement: Supplementary file 1 [file 414_2026_3820_MOESM1_ESM.docx]

**Generative Artificial Intelligence in Forensic Medicine: A Pilot Study on AI-Simulated Medico-Legal Reports in Healthcare Liability Cases**

**Federica Ministeri^1^, Massimiliano Esposito^2^, Martina Francaviglia^1^, Lucio Di Mauro^1^, Grazia Giulia Pantè^1^, Monica Salerno^1^, Francesco Sessa^3,^+, Cristoforo Pomara^1,^+^,^*.**

^1^ Department of Medical, Surgical and Advanced Technologies “G.F. Ingrassia”, University of Catania, 95121 Catania, Italy; [federicaministeri@gmail.com](mailto:federicaministeri@gmail.com) (F.M.), [martinafrancaviglia95@gmail.com](mailto:martinafrancaviglia95@gmail.com) (M.F.); [dr.luciodimauro@gmail.com](mailto:dr.luciodimauro@gmail.com) (L.D.M.); [graziagiuliapante@gmail.com](mailto:graziagiuliapante@gmail.com) (G.G.P.); [monica.salerno@unict.it](mailto:monica.salerno@unict.it) (M.S.); [cristoforo.pomara@unict.it](mailto:cristoforo.pomara@unict.it) (C.P.)

^2^ Faculty of Medicine and Surgery, “Kore” University of Enna, 94100 Enna, Italy; [massimiliano.esposito@unikore.it](mailto:massimiliano.esposito@unikore.it) (M.E.)

^3^ Department of Psychology and Health Sciences - Faculty of Human Sciences, Education, and Sports - Pegaso Telematic University, 80143 Naples, Italy; [francesco.sessa@unipegaso.it](mailto:francesco.sessa@unipegaso.it) (F.S.)

+these authors shared the last position

**Supplementary Material 1** Summary Table of Sources Loaded into the GPTs

| **Title / Authors** | **Description** | **Loaded into** |
| --- | --- | --- |
| Norelli, G. A., Buccelli, C., & Fineschi, V. (2013) | “Medicina Legale e delle Assicurazioni – II edizione (Piccin Nuova Libraria)” | Both GPTs |
| Bolino, G. (2016) | “Linee guida per la valutazione del danno alla persona in ambito civilistico” | Both GPTs |
| Puccini, C. (2003) | Chapter Three: “La causalità” | Both GPTs |
| Marchetti, D., & Fiori, A. (2009) | Chapter Five: “L’evoluzione della giurisprudenza in tema di accertamento del nesso causale” | Both GPTs |
| Di Mauro, L., Rapisarda, G.M., Iannuzzi, S., Dell’Osso, G. (2000) | “Valutazione Medico-Legale del Danno Biologico Temporaneo e Dimensione Storicistica” | Both GPTs |
| Instructions for Hospital report | Specific Instructions for the GPT “AI in Defense of the Hospital” | AI in Defense of the Hospital |
| Instructions for Patient report | Specific Instructions for the GPT “AI in Defense of the Patient” | AI in Defense of the Patient |

**Supplementary Material 2** Summary Table of the Contents of the Customized Instructions

| **GPT** | **Objective of the Report** | **Main Contents of the Instructions** |
| --- | --- | --- |
| AI in Defense of the Hospital | To draft a defensive medico-legal report in favor of the healthcare facility | - Highlight the appropriateness of the clinical management  - Exclude or mitigate profiles of liability  - Assess any biological damage resulting in the patient  - Use clinical guidelines and scientific literature published prior to the event  - Cite articles and protocols with references to PubMed/Scopus  - Structure the report according to a predefined framework |
| AI in Defense of the Patient | To draft a medico-legal report aimed at highlighting any potential healthcare liabilities | - Highlight critical issues, omissions, or errors in clinical management  - Bring to light any potential deviation from standards of care  - Assess any biological damage potentially suffered by the patient  - Use scientific sources and clinical guidelines published prior to the event  - Cite articles and protocols with references to PubMed/Scopus  - Structure the report according to a predefined framework |

**Supplementary Material 3** Complete specific Instructions for the GPT “AI in Defense of the Hospital”

**Supplementary Material 4** Complete specific Instructions for the GPT “AI in Defense of the Patient”

**Supplementary Material 5** Qualitative analysis of the medico-legal considerations drafted by artificial intelligence

| **Cases without established liability on the part of the Hospital** | **Case series** | **Medical–surgical setting** | **Score of the simulated medico-legal report in defense of the Patient** | **Score of the simulated medico-legal report in defense of the Hospital** |
| --- | --- | --- | --- | --- |
|  | Case 1 | General surgery | 1 | 9 |
|  | Case 2 | Gynecology | 10 | 9 |
|  | Case 3 | Urology | 1 | 8 |
|  | Case 4 | Gynecology | 9 | 7 |
|  | Case 5 | Orthopedics | 5 | 6 |
| **Cases with established liability on the part of the Hospital** | Case 6 | Dermatology  (diagnostic error) | 1 | 1 |
|  | Case 7 | Emergency Department (diagnostic error) | 8 | 10 |
|  | Case 8 | Emergency Department/ General surgery  (diagnostic pathway error) | 9 | 2 |
|  | Case 9 | Orthopedics | 7 | 4 |
|  | Case 10 | Oncology (diagnostic error) | 1 | 1 |

**Supplementary Material 6** Qualitative analysis of the clinical summary generated by artificial intelligence

| **Case series** | **Score of the simulated medico-legal report in defense of the Patient** | **Score of the simulated medico-legal report in defense of the Hospital** |
| --- | --- | --- |
| Case 1 | 3 | 3 |
| Case 2 | 3 | 3 |
| Case 3 | 3 | 3 |
| Case 4 | 3 | 3 |
| Case 5 | 2 | 3 |
| Case 6 | 2 | 2 |
| Case 7 | 3 | 3 |
| Case 8 | 2 | 2 |
| Case 9 | 3 | 3 |
| Case 10 | 1 | 1 |

**Supplementary Material 7** Quantitative analysis of bibliographic sources.

| **Case series** | **Reports generated by artificial intelligence** | | **Reports drafted by a human expert** | |
| --- | --- | --- | --- | --- |
|  | **Report in defense of the Hospital** | **Report in defense of the Patient** | **Report in defense of the Hospital** | **Report in defense of the Patient** |
| **Case 1** | 11 | 6 | 9 | 0 |
| **Case 2** | 4 | 3 | 4 | 0 |
| **Case 3** | 4 | 3 | 26 | 0 |
| **Case 4** | 5 | 10 | 9 | 0 |
| **Case 5** | 10 | 7 | 7 | 1 |
| **Case 6** | 4 | 6 | 1 | 1 |
| **Case 7** | 8 | 6 | 1 | 0 |
| **Case 8** | 5 | 5 | 7 | 0 |
| **Case 9** | 3 | 5 | 4 | 0 |
| **Case 10** | 13 | 5 | 2 | 0 |

**Supplementary Material 8** Qualitative analysis of bibliographic sources cited by artificial intelligence

| **Case series** | **Medico-legal report in defense of:** | **Number of scientific citations** | **Authors**  **(0-1)** | **Scientific journal**  **(0-1)** | **Year of publication**  **(0-1)** | **DOI/**  **Link**  **(0-1)** | **Relevance**  **(0-1-2-3)** |
| --- | --- | --- | --- | --- | --- | --- | --- |
| **1** | Patient | 1° | 0 | 1 | 1 | 1 | 3 |
|  |  | 2° | 1 | 1 | 1 | 1 | 3 |
|  |  | 3° | 1 | 1 | 1 | 1 | 3 |
|  |  | 4° | 1 | 1 | 1 | 1 | 3 |
|  |  | 5° | 1 | 1 | 1 | 1 | 3 |
|  |  | 6° | 1 | 1 | 1 | 1 | 3 |
|  | Hospital | 1° | 1 | 1 | 1 | 1 | 3 |
|  |  | 2° | 1 | 1 | 1 | 1 | 3 |
|  |  | 3° | 1 | 1 | 1 | 0 | 2 |
|  |  | 4° | 1 | 1 | 1 | 0 | 2 |
|  |  | 5° | 1 | 1 | 1 | 1 | 2 |
|  |  | 6° | 1 | 1 | 1 | 0 | 3 |
|  |  | 7° | 1 | 1 | 1 | 1 | 2 |
|  |  | 8° | 1 | 1 | 1 | 1 | 3 |
|  |  | 9° | 1 | 1 | 1 | 1 | 3 |
|  |  | 10° | 1 | 1 | 1 | 0 | 3 |
|  |  | 11° | 1 | 1 | 1 | 0 | 2 |
| **2** | Patient | 1° | 1 | 1 | 1 | 0 | 3 |
|  |  | 2° | 1 | 1 | 1 | 1 | 3 |
|  |  | 3° | 0 | 0 | 0 | 0 | 0 |
|  | Hospital | 1° | 1 | 1 | 1 | 0 | 3 |
|  |  | 2° | 1 | 1 | 1 | 1 | 2 |
|  |  | 3° | 1 | 1 | 1 | 1 | 3 |
|  |  | 4° | 1 | 1 | 1 | 1 | 3 |
| **3** | Patient | 1° | 0 | 0 | 0 | 0 | 0 |
|  |  | 2° | 1 | 1 | 1 | 0 | 3 |
|  |  | 3° | 1 | 1 | 1 | 0 | 3 |
|  | Hospital | 1° | 1 | 1 | 1 | 1 | 3 |
|  |  | 2° | 1 | 1 | 1 | 1 | 3 |
|  |  | 3° | 1 | 1 | 1 | 1 | 3 |
|  |  | 4° | 1 | 1 | 1 | 1 | 1 |
| **4** | Patient | 1° | 1 | 1 | 1 | 1 | 1 |
|  |  | 2° | 1 | 1 | 1 | 1 | 2 |
|  |  | 3° | 1 | 1 | 1 | 0 | 3 |
|  |  | 4° | 1 | 1 | 1 | 0 | 3 |
|  |  | 5° | 1 | 1 | 1 | 1 | 3 |
|  |  | 6° | 1 | 1 | 1 | 1 | 3 |
|  |  | 7° | 1 | 1 | 1 | 1 | 3 |
|  |  | 8° | 1 | 1 | 1 | 1 | 2 |
|  |  | 9° | 1 | 1 | 1 | 1 | 3 |
|  |  | 10° | 1 | 1 | 1 | 1 | 2 |
|  | Hospital | 1° | 1 | 1 | 1 | 1 | 1 |
|  |  | 2° | 1 | 1 | 1 | 0 | 3 |
|  |  | 3° | 1 | 1 | 1 | 1 | 3 |
|  |  | 4° | 1 | 1 | 1 | 1 | 2 |
|  |  | 5° | 1 | 1 | 1 | 0 | 1 |
| **5** | Patient | 1° | 1 | 1 | 1 | 0 | 3 |
|  |  | 2° | 1 | 1 | 1 | 0 | 3 |
|  |  | 3° | 0 | 0 | 0 | 0 | 0 |
|  |  | 4° | 0 | 0 | 0 | 0 | 0 |
|  |  | 5° | 0 | 0 | 0 | 0 | 0 |
|  |  | 6° | 0 | 0 | 0 | 0 | 0 |
|  |  | 7° | 0 | 0 | 0 | 0 | 0 |
|  | Hospital | 1° | 1 | 1 | 1 | 1 | 3 |
|  |  | 2° | 1 | 1 | 1 | 1 | 3 |
|  |  | 3° | 1 | 1 | 1 | 1 | 3 |
|  |  | 4° | 1 | 1 | 1 | 1 | 2 |
|  |  | 5° | 1 | 1 | 1 | 1 | 2 |
|  |  | 6° | 1 | 1 | 1 | 1 | 2 |
|  |  | 7° | 1 | 1 | 1 | 0 | 2 |
|  |  | 8° | 1 | 1 | 1 | 1 | 0 |
|  |  | 9° | 0 | 0 | 0 | 0 | 1 |
|  |  | 10° | 1 | 1 | 0 | 0 | 1 |
| **6** | Patient | 1° | 1 | 1 | 1 | 1 | 3 |
|  |  | 2° | 1 | 1 | 1 | 1 | 3 |
|  |  | 3° | 0 | 1 | 1 | 0 | 3 |
|  |  | 4° | 0 | 0 | 0 | 0 | 0 |
|  |  | 5° | 0 | 0 | 0 | 0 | 3 |
|  |  | 6° | 0 | 0 | 0 | 0 | 3 |
|  | Hospital | 1° | 1 | 1 | 1 | 1 | 2 |
|  |  | 2° | 1 | 1 | 1 | 1 | 3 |
|  |  | 3° | 1 | 1 | 1 | 1 | 3 |
|  |  | 4° | 1 | 1 | 1 | 0 | 3 |
| **7** | Patient | 1° | 0 | 0 | 0 | 0 | 0 |
|  |  | 2° | 1 | 1 | 1 | 1 | 0 |
|  |  | 3° | 1 | 1 | 1 | 0 | 3 |
|  |  | 4° | 0 | 0 | 0 | 0 | 0 |
|  |  | 5° | 0 | 0 | 0 | 0 | 0 |
|  |  | 6° | 0 | 0 | 0 | 0 | 0 |
|  | Hospital | 1° | 1 | 1 | 0 | 0 | 3 |
|  |  | 2° | 0 | 0 | 0 | 0 | 0 |
|  |  | 3° | 0 | 0 | 0 | 0 | 0 |
|  |  | 4° | 0 | 0 | 0 | 0 | 0 |
|  |  | 5° | 0 | 0 | 0 | 0 | 0 |
|  |  | 6° | 0 | 0 | 0 | 0 | 0 |
|  |  | 7° | 1 | 1 | 1 | 1 | 1 |
|  |  | 8° | 1 | 1 | 1 | 1 | 1 |
| **8** | Patient | 1° | 1 | 1 | 1 | 1 | 0 |
|  |  | 2° | 1 | 1 | 1 | 1 | 0 |
|  |  | 3° | 1 | 1 | 1 | 1 | 0 |
|  |  | 4° | 1 | 1 | 1 | 0 | 0 |
|  |  | 5° | 1 | 1 | 1 | 0 | 0 |
|  | Hospital | 1° | 1 | 1 | 1 | 1 | 3 |
|  |  | 2° | 1 | 1 | 1 | 1 | 2 |
|  |  | 3° | 1 | 1 | 1 | 1 | 3 |
|  |  | 4° | 1 | 1 | 1 | 1 | 1 |
|  |  | 5° | 1 | 1 | 1 | 1 | 3 |
| **9** | Patient | 1° | 1 | 1 | 1 | 1 | 3 |
|  |  | 2° | 1 | 1 | 1 | 1 | 3 |
|  |  | 3° | 0 | 1 | 1 | 1 | 0 |
|  |  | 4° | 1 | 1 | 1 | 1 | 3 |
|  |  | 5° | 0 | 0 | 0 | 0 | 0 |
|  | Hospital | 1° | 1 | 1 | 1 | 0 | 3 |
|  |  | 2° | 1 | 1 | 1 | 0 | 3 |
|  |  | 3° | 1 | 1 | 1 | 1 | 3 |
| **10** | Patient | 1° | 1 | 1 | 1 | 0 | 1 |
|  |  | 2° | 1 | 1 | 1 | 1 | 2 |
|  |  | 3° | 0 | 0 | 0 | 0 | 0 |
|  |  | 4° | 1 | 1 | 1 | 1 | 1 |
|  |  | 5° | 0 | 0 | 0 | 0 | 0 |
|  | Hospital | 1° | 0 | 0 | 0 | 1 | 3 |
|  |  | 2° | 1 | 1 | 1 | 1 | 3 |
|  |  | 3° | 1 | 0 | 1 | 0 | 3 |
|  |  | 4° | 1 | 1 | 1 | 1 | 3 |
|  |  | 5° | 1 | 1 | 1 | 0 | 3 |
|  |  | 6° | 1 | 1 | 1 | 0 | 1 |
|  |  | 7° | 1 | 1 | 1 | 0 | 0 |
|  |  | 8° | 1 | 1 | 1 | 1 | 0 |
|  |  | 9° | 1 | 1 | 1 | 0 | 0 |
|  |  | 10° | 1 | 1 | 1 | 0 | 3 |
|  |  | 11° | 1 | 1 | 1 | 0 | 2 |
|  |  | 12° | 1 | 1 | 1 | 0 | 0 |
|  |  | 13° | 0 | 0 | 0 | 0 | 0 |

**Supplementary Material 9** Qualitative analysis of the percentages of permanent biological damage estimated by artificial intelligence

|  | **Case series** | **Simulated report in defense of the Hospital** | **Simulated report in defense of the Patient** |
| --- | --- | --- | --- |
| **Cases without established liability on the part of the Hospital** | Case 1 | / | / |
|  | Case 2 | / | / |
|  | Case 3 | / | / |
|  | Case 4 | / | / |
|  | Case 5 | / | / |
| **Cases with established liability on the part of the Hospital** | Case 6 | 3 | 3 |
|  | Case 7 | 2 | 3 |
|  | Case 8 | 0 | 3 |
|  | Case 9 | 0 | 3 |
|  | Case 10 | 0 | 0 |
